# Best practice framework for Patient and Public Involvement (PPI) in collaborative data analysis of qualitative mental health research: methodology development and refinement

**DOI:** 10.1186/s12888-018-1794-8

**Published:** 2018-06-28

**Authors:** Helen Jennings, Mike Slade, Peter Bates, Emma Munday, Rebecca Toney

**Affiliations:** 1 Department of Occupational Therapy, School of Health Sciences, York St. John University, York, UK; 2 School of Health Sciences, Institute of Mental Health, University of Nottingham, Triumph Road, Nottingham, NG7 2TU UK; 3 Peter Bates Associates Ltd, Nottingham, UK; 4 RECOLLECT Lived Experience Advisory Panel, Nottingham, UK

**Keywords:** Patient and public involvement (PPI), Mental health research, Qualitative, Collaborative data analysis, Co-production

## Abstract

**Background:**

Patient and Public Involvement (PPI) in mental health research is increasing, especially in early (pre-funding) stages. PPI is less consistent in later stages, including in analysing qualitative data. The aims of this study were to develop a methodology for involving PPI co-researchers in collaboratively analysing qualitative mental health research data with academic researchers, to pilot and refine this methodology, and to create a best practice framework for collaborative data analysis (CDA) of qualitative mental health research.

**Methods:**

In the context of the RECOLLECT Study of Recovery Colleges, a critical literature review of collaborative data analysis studies was conducted, to identify approaches and recommendations for successful CDA. A CDA methodology was developed and then piloted in RECOLLECT, followed by refinement and development of a best practice framework.

**Results:**

From 10 included publications, four CDA approaches were identified: (1) consultation, (2) development, (3) application and (4) development and application of coding framework. Four characteristics of successful CDA were found: CDA process is co-produced; CDA process is realistic regarding time and resources; demands of the CDA process are manageable for PPI co-researchers; and group expectations and dynamics are effectively managed. A four-meeting CDA process was piloted to co-produce a coding framework based on qualitative data collected in RECOLLECT and to create a mental health service user-defined change model relevant to Recovery Colleges. Formal and informal feedback demonstrated active involvement. The CDA process involved an extra 80 person-days of time (40 from PPI co-researchers, 40 from academic researchers). The process was refined into a best practice framework comprising Preparation, CDA and Application phases.

**Conclusions:**

This study has developed a typology of approaches to collaborative analysis of qualitative data in mental health research, identified from available evidence the characteristics of successful involvement, and developed, piloted and refined the first best practice framework for collaborative analysis of qualitative data. This framework has the potential to support meaningful PPI in data analysis in the context of qualitative mental health research studies, a previously neglected yet central part of the research cycle.

## Background

Patient and Public Involvement (PPI) in research is increasing. PPI can be defined as the involvement of patients, carers and the public as active partners in the design, delivery and dissemination of research to ensure that it is relevant and useful [1], or as “*research being carried out ‘with’ or ‘by’ members of the public rather than ‘to’, ‘about’ or ‘for’ them*” [2]. The impact of PPI on research has been investigated. A 2012 systematic review of PPI in health and social care research identified benefits as enhanced quality and appropriateness of research, development of user-focused research objectives, user-relevant research questions, user-friendly information, questionnaires and interview schedules, appropriate recruitment strategies for studies, consumer-focused interpretation of data and enhanced implementation and dissemination of study results [3]. More recent research has shown positive impacts on researchers, including their knowledge, priorities, lay communication skills and attitudes to involvement [4]. The PPI representative also benefits, e.g. through an improved life focus and relationship with their illness [1]. PPI specifically in mental health research has also shown benefits, e.g. with more PPI found in studies achieving recruitment targets [5].

The level of involvement can differ [6, 7]. Low-level involvement, called ‘informed’, ‘consulted’ or ‘participation’, consists of researchers asking for views which are then used to inform research decision making. Medium-level involvement, called ‘involved’, ‘collaboration’ or ‘co-production’, has a focus on equity within the relationship between the researcher and the PPI participant, and comprises an ongoing partnership with shared decision making. High-level involvement called ‘influential’, ‘control’ or ‘service user-led’, consists of people with experience of the health issue being researched having the dominant voice, delivering and managing research themselves. This framework was chosen as a pragmatic, understandable approach which is measurable [8], relates to mental health areas such as shared decision making [9] and experience of care [10], and is widely used in health research in the United Kingdom. Other frameworks for characterising involvement exist, for example at the individual level (micro level), the health-care service level (meso level), policy level (macro level) and, as in the current study, in research and education (meta level) [11]. Inclusive disability research has highlighted the importance of methods of involvement, including emancipatory research, collaboration research, and, as in this study, steering and advisory groups [12]. Involvement in research can vary across different dimensions; a recent framework published in the United Kingdom identifies six standards: inclusive opportunities, working together, support & learning, communications, impact and governance [13]. A systematic review identified types of PPI in research at the preparatory, execution and translational phases of the research cycle [14].

Applying this three-level involvement framework to clinical services, a movement towards higher levels of service user involvement in clinical practice is evident. For example, shared rather than clinician-led decision-making is now routinely recommended [15], because decision-making involvement influences recovery [16]. Just as in PPI, involvement in clinical decision-making is influenced by relational aspects, including a collaborative and trusting relationship, and access to information resources [17]. User involvement in strengthening services (e.g. in low resources settings [18]) or supporting increased participation (e.g. in in-patient settings [19]) is developing. The growth of peer support workers [20] and the development of service user-run services [21] indicates a trend towards high-level involvement in clinical services. However, the views of mental health staff (especially in-patient and less experienced staff) are more positive about patient involvement in treatment than their involvement in the planning and management of services or in professional education [22]. A systematic review concluded that organisations are still negotiating the balance between consumer leadership and traditional structures and systems [23].

Turning to PPI in research, the level of involvement may not be consistent across all parts of the research cycle. An important driver of behaviour in the scientific research community is funding, and many funders now require PPI input to proposals. For example, the leading funder of applied health research in the UK is the National Institute of Health Research (NIHR), and NIHR has a stated expectation that there will be active involvement of members of the public in the research that it funds [24]. This is operationalised through mandatory PPI sections in proposal forms for applicants to outline the extent of PPI, and through PPI representatives on funding panels. As a result, PPI in the UK has become the norm in developing health research proposals.

PPI involvement after securing funding is inconsistent. Examples of token involvement during post-funding research stages (study setup, ethical approval, data collection, data analysis, dissemination) are reported, such as more importance being given to researcher and clinician views than to PPI perspectives, and PPI processes being experienced as a tick-box exercise [25, 26]. Whilst the public voice is increasingly present in research decision-making, there is less evidence of a change in the underpinning power dynamic between the scientific research community and the public [27]. Mirroring the stage of development of involvement in clinical services (outlined earlier), the transition towards citizen science – equal partnership between scientists and citizens – is only partially complete [28].

As part of the movement towards higher levels of PPI in academic research, analysis of data is an important point of input [29]. However, service users are rarely involved in creating meaning from data [30]. This means that a valuable perspective in interpreting findings is being lost. Collaborative data analysis (CDA) of qualitative data is a recognised methodology involving a joint focus and dialogue among two or more researchers regarding a shared body of data to produce an agreed interpretation [31]. When one of the involved parties bring a PPI perspective, this can highlight taken-for-granted researcher assumptions, provide socio-cultural and political insights, and enhance the thoroughness of interpretation [32]. For example, in a study of detained psychiatric patients, mental health service user researchers coded more according to experiences and feelings, whereas university researchers coded more according to procedures and processes [33].

The Recovery Colleges Characterisation and Testing (RECOLLECT) Study is an NIHR-funded evaluation of Recovery Colleges (RCs), a new innovation which uses co-production and adult education approaches, rather than treatment, to support mental health recovery [34, 35]. RCs involve the development of a culture of ‘emancipatory education’ [36] with an emphasis on ‘inclusivity and egalitarianism’ [37]. Emerging fidelity criteria are located in counter-point to the current mental health system [38]. As part of RECOLLECT, qualitative data on mechanisms of action and outcomes were collected in three RCs. As RCs are a disruptive innovation [39] based on intentionally different values, goals and assumptions from clinical systems, there is a high risk of bias in interpreting qualitative data about them from a clinical research perspective. Therefore a collaborative data analysis approach involving people with lived experience was required to improve the quality of analysis. We refer to the (typically university-based) academic research team conducting studies (such as RECOLLECT) as the **academic researchers** (whilst recognising that academic researchers may themselves have disclosed or non-disclosed experience of mental ill-health, and noting the existence of service user-led research groups in universities) and people bringing a public or patient perspective as **PPI co-researchers** (also noting that they may have research experience).

The aims of this study were (1) to develop a methodology for involving PPI co-researchers in analysis of qualitative data; (2) to pilot and refine this methodology; and (3) to create a best practice framework for future PPI in data analysis.

## Methods

The RECOLLECT Lived Experience Advisory Panel (LEAP) comprises a group of 9 people with lived experience of mental health issues, either personally or as carers. Members brought a range of research experience (from never having done research before to having collaborated on several projects), and were heterogeneous in where they lived, educational achievement and occupational background. LEAP members are referred to as the PPI co-researchers.

The RECOLLECT research team (who collected the data collaboratively analysed in Stage 2) span a range of professions (counselling, occupational therapy, psychology) and PPI experiences (PPI lead, PPI participant), and specifically include people with and without lived experience of mental health issues (directly or as a family member). The RECOLLECT research team are referred to as the academic researchers.

Ethical approval was obtained (Nottingham REC 1, 16/EM/0484) and all participants provided written informed consent.

### Stage 1: Develop the CDA methodology

A critical literature review [40] was conducted by HJ, involving evaluation and synthesis of included papers. The aim of a critical review is to identify the most significant articles in a field, and it was chosen over more systematic approaches as a proportionate review approach suitable for generating a model. Databases searched were AMED, PubMed, CINAHL, MEDLINE and PsycARTICLES, with serendipitous searching [41] to identify grey literature. A scoping search indicated that terms like ‘coproduction’ were insignificantly specific, so search terms specifically focussed on the collaborative analysis element of coproduction were used. Inclusion criteria were (a) primary focus on qualitative collaborative data analysis; (b) published in journal or book; (c) focused on mental health research; (d) aligned with the principles of PPI and a democratic approach to public engagement [7]; and (e) published since 2007 to maximise relevance to current PPI frameworks. The initial concept search strategy comprised (“collaborative data analysis” OR “interpretation workshop” OR “participatory action research”) AND (“service user” OR “public patient involvement” OR “PPI” OR “co-researcher” OR “co-investigator” OR “expert by experience” OR “client” OR “consumer” OR “survivor”) AND (“mental health” OR “mental illness” OR “mental distress” OR “psychological distress”) AND “qualitative”. The initial search identified few papers specific to mental health, so inclusion criterion (c) was amended to include any health research, and the search strategy amended accordingly. The first author extracted data relevant to design and procedure, and characteristics of successful CDA. Findings were synthesised by the research team, and organised into methodology options and success characteristics.

### Stage 2: Pilot and refine the CDA methodology

The Stage 1 synthesis was considered by academic researchers and PPI co-researchers to select a methodology for CDA in RECOLLECT, in relation to investigation about mechanisms, outcomes and change models for Recovery Colleges. Evaluation criteria were that the methodology: ensured PPI co-researcher interpretations of qualitative data collected in RECOLLECT were generated; ensured these interpretations were used to inform findings; aligned with a democratic approach to PPI [7]; were practical to apply within the constraints of a one-year study; and incorporated the design techniques that were associated with successful CDA. Decisions were amalgamated by HJ into a final synthesised methodology and procedural session plans, which were then refined by the other authors. The CDA methodology was then piloted. PPI co-researchers met with the academic researchers in four 4-h meetings, each with a minimum of 8 co-researchers present who were paid for their preparation and attendance.

The aim of this part of RECOLLECT was to develop an understanding of how RCs work (mechanisms of action) and the impact on students (outcomes). Framework analysis [42] was the underpinning methodology. Summarising the content of CDA meetings (described in Table 3): Meeting 1 ensured the ecological validity of the document analysis carried out by the academic researchers; Meeting 2 refined the preliminary, academic researcher-developed coding framework to co-produce the final coding framework; Meeting 3 created a mental health service user-led change model (organising and linking coded mechanisms of action and outcomes); and Meeting 4 enabled final verification of the change model. Each meeting was facilitated by two RECOLLECT academic researchers; one to help navigate the narrative of the discussions and the other to support group dynamics and ensure all PPI co-researchers were able to contribute. Tasks were predominantly broken down into small group or pair work initially, with all PPI co-researchers then coming together to share what they had produced. Exercises were presented both verbally and on paper, with opportunities for questions and challenge. Adequate understanding of task requirements was ascertained through check-back and observation. Insights from occupational therapy research [43, 44] were used to inform grading and adapting activities to align with an individual’s capacities, in order to maximise their performance. This improves the likelihood the PPI co-researcher experience will be positive, rather than frustrating, marginalising or demoralising [45]. Exercises were paced to allow time for breaks and refreshment. Data interpretations by PPI co-researchers were captured by two academic researchers observing and writing down PPI co-researcher verbal contributions, collection of written and photographed outputs, and completion of field notes by the academic researchers.

Several strategies to enhance quality were used, such as peer examination, triangulation of researchers and PPI co-researcher perspective, triangulation of data sources (PPI, documents, qualitative interviews), and use of reflexivity in the academic researcher field notes [46]. Additionally, in the final meeting informal feedback was obtained and the Quality Involvement Questionnaire was completed by PPI co-researchers [47]. This 31-item self-report measure (each item rated 0 (low involvement) to 4) assesses perceived involvement, and has six sub-scales in two parts: Personal Factors (Your ability (range 0–28), Your potential (0–20), Your sense of being (0–20)) and Research Contexts (Research relationships (0–24), Ways of doing research (0–16) and Research structures (0–16)). Total score ranges from 0 (low involvement) to 124. As normative data are not available, it was also completed by the academic researchers to provide a comparison group.

### Stage 3: Best practice framework for CDA

The most useful components of the methodology were synthesised by the academic researchers to produce best practice, based on data collected during Stage 2.

## Results

### Stage 1: Develop the CDA methodology

The critical review identified 10 publications for inclusion, shown in Table 1 [30–33, 48–53].

**Table 1:** Included publications (*n* = 10)

Ref.	CDA approach	Qualitative design	CDA related findings
[30]	Investigates the value of multiple coding in CDA	Case study	The team were able to develop a strong consensus on the data utilising multiple perspectives
[48]	Qualitative document analysis of NIHR PPI	Case study	Involving members of the public in analysis was successful
[32]	Co-research with people with learning disabilities	Reflective report on ethnographic study	People with learning disabilities can be co-researchers with appropriate support, time and financing
[31]	Methodology chapter exploring CDA theory and application	N/A	N/A
[33]	Investigates service users interpretations of qualitative data	Secondary analysis of papers coded by PPI vs. non-PPI researchers	Service user researchers brought a different perspective, coding according to experiences and feelings, whereas university researchers coded according to processes and procedures
[49]	Process and outcomes of involving service users in data analysis.	Case study	Developed a methodology for conducting long term CDA with people with life limiting conditions
[50]	Process of involving service users in CDA	Case study	Analysed the benefits and challenges of doing CDA with people exploring involvement of patients in medication safety
[51]	Investigates the value of multiple perspectives when interpreting transcripts.	Case study	Service user researchers enhanced the breadth and depth of findings, improving overall study quality
[52]	Describes service user involvement in data analysis	Participatory study	Identified the value of the service users in sharing their perspective in CDA
[53]	Describes involving people with mental health issues in long term CDA	Case study	Described a methodology for conducting long term CDA with people with mental health issues. Found that lack of service user input in early stages of the project impacted on the extent to which co-production was achieved

One publication was a methodology chapter [31], and the others were empirical studies using qualitative methodologies: thematic analysis [30, 33, 49, 52], qualitative content analysis [33, 48], interpretative phenomenological analysis [51], grounded theory [32] and framework analysis [48, 50, 53]. There was strong consensus across all studies that including co-researchers with lived experience in qualitative research data analysis produced richer, more in-depth and alternative understandings of the material that the academic researchers could not have developed on their own. Four methodological approaches to involving PPI co-researchers in data analysis were identified.

#### CDA approach 1: Consultation

In this approach the academic researchers conducted the analysis process and then presented their work to PPI co-researchers for commentary and feedback. Points of ambiguity or non-consensus identified by academic researchers were highlighted to PPI co-researchers, to achieve a PPI-informed perspective on the data. Studies using this approach tended to have people with lived experience in their research team [30, 33], so while this approach may appear the least democratic CDA approach, these studies had moved beyond the binary categorisation of researchers as academic or service users to reflect the reality of multiple aspects to identity which influence data interpretation and reflexive understanding. When used in studies with this mix of researchers in the academic team, it provides a rapid and cost-effective approach to involving a wider range of PPI perspectives.

#### CDA approach 2: Development

In studies using this approach [48, 51], PPI co-researchers were involved in the early stages of analysis, inductively developing themes, codes or frameworks based on small samples of transcripts. These were then deductively applied by the academic researchers to the rest of the data, with resulting points of ambiguity revisited with the PPI co-researchers for clarification and commentary. Hence the focus was on PPI input to create the fundamental constructs informing how the data are understood, with the more procedural work of applying these constructs to a wider range of transcripts delegated to academic researchers. This approach fits projects with financial and time constraints, where achieving maximum PPI input with minimum expenditure is needed.

#### CDA approach 3: Application

In this approach, the academic researchers lead the analysis process, and then involve PPI co-researchers in applying themes, codes and frameworks to a range of transcripts, including consultation on areas of ambiguity and non-consensus [50]. PPI co-researchers do not create the constructs they are applying to the data, so (unless people with lived experience are in the academic team) this does not lead to service user input to how the data are understood.

#### CDA approach 4: Development and application

In studies using this approach [49, 52, 53], PPI co-researchers are given extensive training in data analysis techniques, hold multiple meetings over extensive time periods to thoroughly interrogate the data and achieve meaning at a deep, semantic level, and undertake extensive co-revision of themes, codes and frameworks as findings emerge. In essence, if the academic researchers are doing it, so are the PPI co-researchers. The process is entirely co-produced at all levels, so this approach might be termed the gold standard for PPI co-researcher involvement in CDA. In studies with long time scales and larger budgets, this is the most democratic approach to use.

#### Characteristics of successful CDA

Four characteristics of successful CDA were identified, shown in Table 2 with illustrative texts.

**Table 2:** Four characteristics (with examples) of successful Collaborative Data Analysis

1. The CDA process is co-produced	
• Keep consulting; verify everything with PPI co-researchers [48] • Good facilitation with a supportive and valuing approach is essential. Understand the perspectives/positions of the PPI co-researchers interpreting the data. Be reflexive Be mindful of the personal investments people can hold in how topics are interpreted. [31] • Support PPI co-researchers to understand that while experience can be used to help interpret the data, all interpretations must have some basis in that data [50] • Ensure a range of perspectives amongst the PPI co-researchers interpreting the data; aim for a heterogeneous group [31, 50] • Listen to and explore differences of opinion. When non-consensus occurs, try to create novel synthesis to acknowledge the range of perspectives [31]	
2. The CDA process is realistic within available time and resources	
• Ensure sufficient resources exist, e.g. time and money to organise and facilitate CDA. Do not underestimate this [31] • Keep the number of analysts relatively small [30]. Use software packages to investigate inter-coder reliability if not everyone is coding all data [31] • Make sure data handling and organisation is meticulous [31]	
3. The demands of the CDA process are manageable for PPI co-researchers	
• Give PPI co-researchers material to read in advance [48, 49]. Make sure materials are accessible and in a range of formats where required [49, 50] • Provide training that ensures people can successfully complete the CDA they have been asked to do [49, 50]. Do ‘warm up’ activities that align with the CDA tasks people are being asked to undertake [48]. Use practical, visual aids like post-its and flip chart paper to support analysis tasks [48, 49] • Keep the data set relatively small and do not present people with too much raw data [30, 48]. Ensure the data analysis process is adjusted to take into account the strengths and needs of PPI co-researchers and is ‘failure free’ [32]. Allow ample time for analysis [30]	
4. Group expectations and dynamics are effectively managed	
• Clearly set out the PPI co-researcher role and expected time commitment [31, 49, 50], and how their contributions will be valued and incorporated [31]. Clarify the division of labour (in writing if appropriate) [31, 49] • Be mindful of labelling: people hold multiple identities and categorisation can cause inter-group tensions. Be vigilant for power imbalances, which may occur even with the best of intentions [31]	

### Stage 2: Pilot and refine the CDA methodology

Approaches 1 and 2 were integrated for use in RECOLLECT, as shown in Table 3. As some of the academic researchers were people with dual identities as researchers and with lived experience, concerns around tokenism in Approach 1 were less relevant. Combining this with Approach 2 allowed more meaningful involvement in understanding the data. The intention was that this combination would offer an effective way of conducting CDA within the time and resource boundaries of the study. Approach 3 was not used as it was least collaborative, and Approach 4 was not feasible in a one-year study. The authors incorporated success characteristics shown in Table 2 into planning of the CDA meetings. The CDA focus and the content of each meeting are detailed below.

**Table 3:** Overview of RECOLLECT CDA sessions

Meeting 1 April 2017	Preparation (Approach 1) Discussion of the aims of the RECOLLECT project. Explanation of the PPI co-researcher role. Joint agreement on role expectations and ground rules (e.g. confidentiality). Identification of initial PPI suggestions about mechanisms and outcomes for RC students for inclusion as a priori codes in preliminary coding framework.
After Meeting 1	Generation of preliminary coding framework by inductive document analysis (*n* = 10) by academic researchers, incorporating meeting 1 suggestions as a priori codes. Circulation of meeting 2 documents (including preliminary coding framework) in advance
Meeting 2 July 2017	Consultation on a coding framework (Approach 1) Education around qualitative research. Reflexive exercise on what PPI co-researchers bring to the analysis. PPI co-researcher commentary on the content and language used in the preliminary coding framework, and on interpreting areas of ambiguity in data informing the preliminary coding framework
After Meeting 2	Contributions regarding content, language and interpreting areas of ambiguity incorporated into the coding framework by the academic researchers. Deductive coding of remaining documents (*n* = 34) and refinement of coding framework by academic researchers. Refined coding framework circulated to PPI co-researchers for feedback. Circulation of all meeting 3 documents in advance
Meeting 3 September 2017	User-led development of a model of change (Approach 2) Re-visiting and finalising of the refined coding framework. Education around models of change and how to create them. PPI co-researchers develop a model of change using components of the coding framework, illustrate the model with practical examples, and identify the most important components
After Meeting 3	Model of change formatted by the academic researchers. Model of change sent to PPI co-researchers for further commentary and refinement. Incorporation of feedback into the model by the academic researchers. Completion and analysis of semi-structured interviews with stakeholders (*n* = 33) by academic researchers to refine the change model. Circulation of meeting 4 documents in advance
Meeting 4 December 2017	Dissemination, reflection, group processing and celebration Final verification of the model of change. Exploring and reconciling (where possible) remaining areas of ambiguity. Completion of Involvement Questionnaire. Celebration of PPI co-researcher achievements

The Quality Involvement Questionnaire was completed by PPI co-researchers (*n* = 6) and academic researchers (n = 6). Self-rated involvement was higher for PPI co-researchers for Your ability (21.2 vs. 20.5), Your potential (15.3 vs 14.2), Research relationships (22.3 vs. 21.0), Ways of doing research (15.7 vs. 14.3) and Research structures (13.2 vs 11.0), and higher for academic researchers for Your sense of being (18.2 vs. 17.5). Total score was 105.2 (84.8%) for PPI co-researchers and 99.2 (80.%) for academic researchers.

Informal feedback was broadly positive, with one suggestion to use more action-focussed, concrete and illustrative tasks, rather than giving too much information about theory. For example, demonstrating the use of string and self-adhesive arrows to show a causal relationship between a mechanism card and an outcome card if the PPI co-researcher believes the mechanism produces the outcome.

### Stage 3: Best practice framework for CDA

Two process findings emerged from conducting the Stage 2 CDA, and from reflections from the involved academic researchers and PPI co-researchers. First, academic researcher facilitators felt that more time was needed for PPI co-researchers to complete the CDA tasks, especially regarding areas of ambiguity and achieving rationalisation and reconciliation in the analyses. Second, a balance was needed between over-planning sessions (risking stifling creativity and encouraging PPI co-researcher passivity) and carefully planning the structure and content of sessions so as to generate the deepest and most collaborative analysis. On balance, we concluded that CDA sessions need to be more structured and formalised than general PPI advisory meetings.

**Fig. 1:**
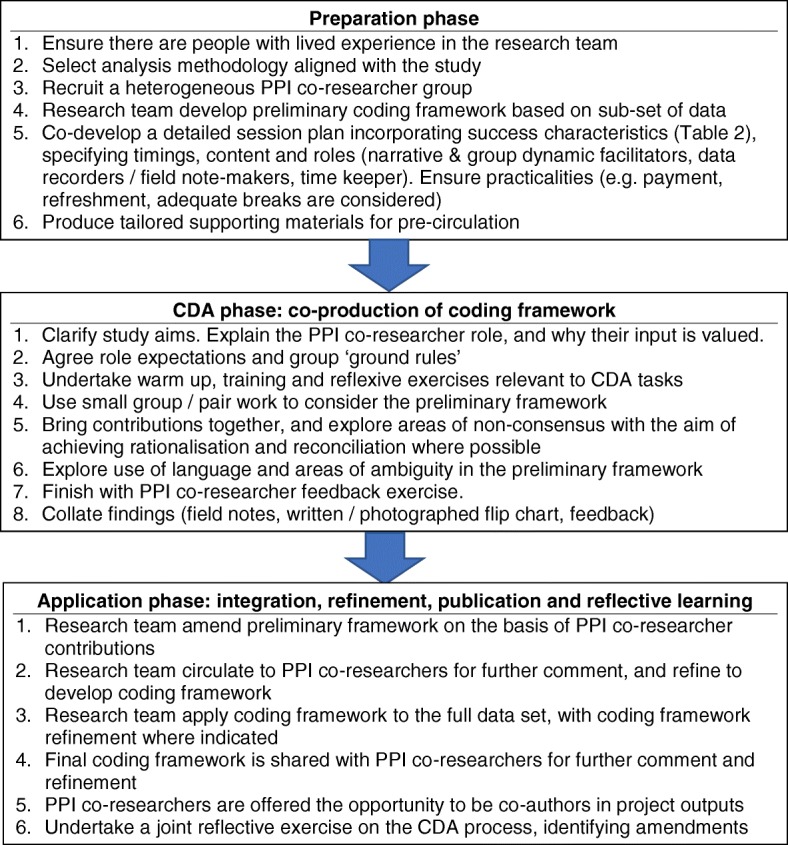
Best practice framework for collaborative data analysis involving people with lived experience in coding framework co-production

Based on this pilot, and for studies with limited time and financial/human resources (i.e. most funded studies, where compromises between quality and pragmatism are required), we identify a three-stage approach to CDA: Preparation, CDA and Application. Figure 1 shows a proposed best practice framework using these three stages to co-produce a qualitative coding framework.

In RECOLLECT we also used these stages to develop a service user-led model of change, developed by the PPI co-researchers and based on the co-produced coding framework. The Preparation phase was unchanged. In the CDA phase the small group work focussed on how model components (from the co-produced coding framework) relate to each other, using active and practical methods (e.g. laminated cards, sticky tape, string, self-adhesive arrows) to facilitate creativity and easy adjustments. In the Application phase a formatted version of the change model was validated with PPI co-researchers, then verified by the academic researchers with new research participants before being finalised with PPI co-researchers.

## Discussion

In this study we reviewed current evidence to identify four reported approaches to CDA involving PPI co-researchers and four characteristics of successful CDA studies. We used this theory base to develop, pilot and refine a methodology, which informed our proposed best practice framework for collaborative analysis of qualitative data in mental health research.

### PPI in collaborative data analysis

The National Involvement Partnership has developed the 4Pi national involvement standards: principles (respect, equality); purpose (potential and limits of change), presence (at all decision-making levels), process (engagement, communication, support, practical issues) and impact (ethos/culture, policy/practice, delivery, outputs/outcomes, diversity and equality of opportunity, PPI experience) [54]. Similarly, INVOLVE published a PPI values and principles framework emphasising respect, support, transparency, responsiveness, fairness of opportunity and accountability [55]. These principles informed RECOLLECT, although the aim of this study was to incorporate these principles whilst recognising the challenges of time-limited studies. If resources were less limited, desirable extensions would be involvement of PPI co-researchers in all elements of the data analysis, training and supporting PPI co-researchers to undertake the integration activities, and involvement (e.g. through online consultation) of a much larger heterogeneous and more representative PPI group. The decision to have the academic researchers develop the preliminary framework was made because the challenges of having PPI co-researchers undertake primary data coding using qualitative analysis software were deemed too complex. The potential bias introduced by this decision in the overarching interpretation could have been reduced by involving a sub-group of PPI co-researchers at the data coding stage.

The review identified only ten studies of PPI in analysis of qualitative data in mental health research. Despite the increasing PPI literature, empirical studies on involvement at this critical research stage of making sense of qualitative data were relatively few, necessitating extension of the search strategy to include non-mental health research studies. There is more published research about PPI in other research stages, such as the early stage of prioritising important research questions [56, 57]. Established methodologies now exist, such as James Lind Alliance priority setting partnerships [58]. PPI impact assessment studies consistently recommend that PPI needs to occur throughout all research stages. For example, in relation to PPI in quality improvement, guidance concludes ‘*To be effective, PPI should run through the full cycle of every quality improvement project, as an integral part of the fabric*’ [59] (p. 26). The absence of methodologies for collaborative data analysis is an important knowledge gap.

Four approaches to CDA were identified. Although they broadly move from lower (Approach 1) to higher (Approach 4) involvement, they cannot be simplistically allocated to a level of involvement. For example, in RECOLLECT the academic researchers included people with a range of lived experience, several study applicants self-identified as having lived experience, and the PI has published and been influenced by PPI impact assessment studies [60, 61]. Similarly, the PPI group had members with a range of research experience. A particular challenge was for academic researchers to avoid viewing PPI co-researchers as ‘just’ bringing a lived experience perspective – the strategic essentialism issue of reifying ‘people with lived experience’ as an undisputed and cohesive category [62]. PPI co-researchers cannot represent the full range of lived experience, and they all bring lived experience as one identity component among many.

Both collaborating parties therefore included members with hybrid identities [63]. Given this complexity, even a consultation approach may be effective, depending on the relational context, which from a PPI perspective is central [64]. The experience of PPI co-researchers will be influenced by whether they trust the academic researchers, whether they perceive the academic researchers really do want their views, even if challenging, and whether changes happen in response to PPI input. The best practice framework adheres to democratic principles, by involving people within research related to their health needs to produce a mutually agreed interpretation of the data [27]. Collaboration was increased by the involvement of people with lived experience as academic researchers, the ongoing relationship between the academic researchers and the PPI group (allowing trusting relationships to form), and repeated contribution verification and incorporation of PPI input [2].

### Evaluating the costs and benefits of CDA

Qualitative paradigms encourage researchers to focus on what being human is like, how people understand their worlds and the ‘lived experience’ [65]. Including PPI co-researchers in analysis supports this goal. Evidence from our pilot confirms that PPI co-researchers interpret data through an overlapping but different lens from academic researchers, so are a resource for highlighting assumptions and reducing interpretive bias [31, 32]. For example, in RECOLLECT the PPI co-researchers proposed that one particular mechanism of change (related to environment) was a precursor to all others in the change model, which was a new – and incorporated – proposal to the academic researchers. This may indicate that a deep understanding of the importance of the social, relational and environment is more easily available to PPI co-researchers than to academic researchers. As a second example, the preliminary framework included a code relating to identity change, but PPI co-researchers highlighted the implicit though unintended implication of ‘moving from bad to good identity’. This may reflect encultured clinical research beliefs about improvement meaning transition from sickness to health. Again, the framework was changed to reflect this insight, and researchers also grew in self-awareness about their own values and assumptions. Overall, our findings were consistent with existing evidence [4] that PPI involvement in collaborative data analysis improves quality of the findings and beneficially impacts on academic researchers. The same finding emerges from ‘inclusive research’ – involving people with intellectual disabilities in research – that acknowledging differences and uniqueness enriches research outcomes and supports reflective practice by researchers [66].

An average of four to five days of academic researcher time was spent in planning each meeting, which involved ensuring that meeting tasks created a ‘just right’ activity challenge [37], developing training approaches that incorporated andragogic principles [67, 68], and developing supporting materials and pre-circulating these to PPI co-researchers for absorption, comment and refinement. Along with the involvement of other academic researchers (*n* = 4) and PPI co-researchers (*n* = 9) in the four whole-day meetings, overall around 20 person-days were used per meeting, i.e. around 80 person-days extra time was involved in collaborative rather than academic researcher-led data analysis. This reinforces previous warnings not to underestimate the scale of resources involved in CDA [31], and is necessary given the challenges of collaborating on complex mental health topics [69].

CDA studies identified in the review did not emphasise the importance of adjusting tasks to build on PPI co-researcher strengths and reduce the impact of needs, other than practical suggestions such as pre-circulating accessible material, allowing time for tasks, not overwhelming PPI co-researchers with data, and using aids like post-its [30, 48–50].

The goal in RECOLLECT was for the collaborative data analysis to inform the interpretation of the findings. There was little in the published literature about how to measure the extent to which this specific goal was met, beyond the recommendation of verifying findings with PPI co-researchers [48] and obtaining feedback [2, 7]. Available guidance is more general, e.g. PPI reporting guidelines [70] or ethical frameworks [25]. There is also a considerable literature on overarching frameworks for involvement in research, both relating to psychosis [71] and wider frameworks such as community-based participatory research [72], which focus more on principles and examples than on developing a best practice framework as in this study. Future research could: (a) develop a standardised measure of CDA impact (including both costs and benefits); (b) refine through piloting and then manualise the best practice framework, including resources such as relevant warm-up tasks and training exercises and potentially incorporating elements from CDA approaches 3 and 4; and (c) conduct a PPI impact assessment to evaluate the impact of CDA on study findings. This would inform decision-making in studies about whether the benefits of collaborative data outweigh the costs.

### Strengths and limitations

This study has developed the first theory-based and piloted best practice framework for collaborative analysis of qualitative data. Strengths include the use of a wide range of health research evidence to inform the framework, the significant investment of human resources, the repeated respondent validation, and the collection of formal and informal feedback demonstrating active involvement by PPI co-researchers.

The findings could be strengthened by conducting a systematised rather than critical review [40], with a stronger focus on obtaining grey literature, use of broader search terms (e.g. coproduction, co-design, emancipatory research, inclusive research, partnership research) and a more formal deductive analysis approach. More consistent involvement of a sub-group of PPI co-researchers in planning the meetings would have increased co-production in the process, and might for example have led to a more structured assessment of accommodations needed by individual PPI co-researchers to allow them to fully contribute. Further piloting, especially in studies which are not as positive towards the use of lived experience as RECOLLECT, would helpfully refine and extend the best practice framework.

## Conclusions

This study has developed a typology of approaches to collaborative data analysis in mental health research, identified from available evidence the characteristics of successful involvement, and developed, piloted and refined the first best practice framework for collaborative analysis of qualitative data. Involvement in analysis of findings can be viewed as a human right [73], and this framework has relevance to any qualitative research study which aims to involve people with experience of mental ill-health in interpreting findings.

## Abbreviations

CDA: Collaborative data analysis
NIHR: National Institute for Health Research
PPI: Patient and Public Involvement
RC: Recovery College
RECOLLECT: Recovery Colleges Characterisation and Testing
